# Global Repression of Host-Associated Genes of the Lyme Disease Spirochete through Post-Transcriptional Modulation of the Alternative Sigma Factor RpoS

**DOI:** 10.1371/journal.pone.0093141

**Published:** 2014-03-26

**Authors:** Daniel P. Dulebohn, Beth M. Hayes, Patricia A. Rosa

**Affiliations:** Laboratory of Zoonotic Pathogens, Rocky Mountain Laboratories, Division of Intramural Research, National Institute of Allergy and Infectious Diseases, National Institutes of Health, Hamilton, Montana, United States of America; University of Toledo School of Medicine, United States of America

## Abstract

*Borrelia burgdorferi*, the agent of Lyme disease, is a vector-borne pathogen that transits between *Ixodes* ticks and vertebrate hosts. During the natural infectious cycle, spirochetes must globally adjust their transcriptome to survive in these dissimilar environments. One way *B. burgdorferi* accomplishes this is through the use of alternative sigma factors to direct transcription of specific genes. RpoS, one of only three sigma factors in *B. burgdorferi*, controls expression of genes required during tick-transmission and infection of the mammalian host. How spirochetes switch between different sigma factors during the infectious cycle has remained elusive. Here we establish a role for a novel protein, BBD18, in the regulation of the virulence-associated sigma factor RpoS. Constitutive expression of BBD18 repressed transcription of RpoS-dependent genes to levels equivalent to those observed in an *rpoS* mutant. Consistent with the global loss of RpoS-dependent transcripts, we were unable to detect RpoS protein. However, constitutive expression of BBD18 did not diminish the amount of *rpoS* transcript, indicating post-transcriptional regulation of RpoS by BBD18. Interestingly, BBD18-mediated repression of RpoS is independent of both the *rpoS* promoter and the 5’ untranslated region, suggesting a mechanism of protein destabilization rather than translational control. We propose that BBD18 is a novel regulator of RpoS and its activity likely represents a first step in the transition from an RpoS-ON to an RpoS-OFF state, when spirochetes transition from the host to the tick vector.

## Introduction

Many vector-borne pathogens are maintained in a natural infectious cycle in which they transition between specific vectors and susceptible hosts. During this vector->host->vector cycle, pathogens are exposed to disparate environments, to which they must quickly adapt through immediate changes in gene expression to ensure successful transmission and acquisition. One such vector-borne pathogen, and the causative agent of Lyme disease, is the spirochete *Borrelia burgdorferi* [1]–[3]. *B. burgdorferi* is transmitted by the bite of infected *Ixodes* ticks and maintained in an enzootic cycle between ticks and small mammalian hosts [4]. Larval *I. scapularis* ticks acquire the pathogen by feeding on an infected host. Spirochetes survive through the molt from larvae to nymph, and are subsequently transmitted to a new host by tick feeding. The spirochetes establish a persistent infection in the host, completing the infectious cycle and making them available for acquisition by feeding ticks [5]–[7].

Throughout the infectious cycle, spirochetes are exposed to difficult environmental conditions, including acquired and innate immune pressures, oxidative and nitrosative stress, and nutrient limitation [8]–[14]. To survive within and transit between these environments, *B. burgdorferi* must quickly and effectively adjust its transcriptome. Characteristic examples of changes in gene expression during the *B*. *burgdorferi* infectious cycle include the timely and critical expression of *ospC, dbpA,* and *bba66* early in mammalian infection [15]–[21], as well as the expression of *ospA* and *glpD* in the tick vector [20], [22]–[25]. While the specific functions of some of these factors are unknown, they have demonstrated roles in the in vivo fitness of *B. burgdorferi* [19]–[21], [25]–[31]. Inappropriately timed or unregulated expression [12], [32]–[34] of some key virulence-associated factors can lead to spirochete clearance from the host, or the inability to survive in the vector. The ability to adapt to environmental changes is requisite for successful transmission of the spirochete; precise control and proper timing of gene expression is critical for survival of the spirochete throughout its infectious cycle.

Control of gene expression in bacteria is complex and occurs through many different molecular mechanisms. One of those mechanisms is the coordinated control of sigma factor-directed transcription. In response to environmental conditions, growth phase or cellular stresses, specific sigma factors become available, bind to the RNA polymerase holoenzyme (RNAP), and direct the transcription of genes required to adapt to particular environments. When the stress is removed, bacteria switch sigma factors, thereby remodeling the transcriptome, to adapt to changing conditions (For review see-[35]). Reprogramming of gene expression through sigma factor-directed transcription, and the use of alternative sigma factors are vital to the success of many pathogens [36], [37]. *B. burgdorferi* accomplishes this during the infectious cycle using only three sigma factors, *rpoD* (σ70), *rpoN* (σ54), and *rpoS* (σ38) [38], [39]. Both RpoN and RpoS play critical roles in the *B. burgdorferi* infectious cycle and, interestingly, RpoN controls the majority of *rpoS* transcription [40]–[44].

In *E. coli* RpoS controls the stress response [45], but in *B. burgdorferi*, as in many other pathogenic bacteria, RpoS controls the transcription of several virulence factors, including *ospC* and *bba66* [44], [46]. OspC and BBA66 are critical for establishing infection when spirochetes are transmitted from a tick to a mammalian host [21], [28], [47], and *rpoS* appears to be maximally expressed during this transmission stage [48], [49]. RpoS, or an RpoS-dependent factor, also plays a central role in the repression of genes that have important roles in the arthropod vector, including *ospAB*, *bba62* and *bba74* [48], [50], [51]. Therefore, proper expression and repression of *rpoS* is crucial throughout the infectious cycle; inappropriately timed expression of OspC or repression of OspA would be detrimental to the survival of *B. burgdorferi*. Consequently, *B. burgdorferi* exerts tight control over *rpoS*, using transcriptional and translational activators and transcriptional repressors [43], [52]–[61]. The regulation of RpoS in *B*. *burgdorferi* is complex [60]. RpoS is requisite for expression of critical virulence factors, is fundamental to establishing an infection in a mammalian host, and must be repressed to allow expression of genes required when spirochetes transition from the mammalian host into the tick.

Linear plasmid 17 (lp17) of *B. burgdorferi* encodes a protein, BBD18, that can repress expression of *ospC* [62]. However, expression of *ospC* is RpoS-dependent, and induction of RpoS-dependent gene transcription requires the activation of a multistep signaling cascade [42], [43], [60], [63], [64]. Additional control of *ospC* expression is also exerted through inverted repeat (IR) sequences located upstream of the *ospC* promoter [33], [65]–[67]. BBD18 is a small (25.7kDa), basic protein that contains sequence determinants suggestive of a role in nucleic acid binding, but where BBD18 is exerting its regulatory effect leading to *ospC* repression was previously undetermined. Here we report that BBD18-mediated repression is not limited to *ospC* and that BBD18 is in fact a novel regulator of RpoS, exerting its effect at a post-transcriptional level, and therefore repressing the entire RpoS regulon. We demonstrate that repression of RpoS is not a result of inhibition of translation initiation, or mediated through the *rpoS* ribosome binding site or the *rpoS* 5’ untranslated region (UTR). Our data suggest that BBD18 plays a role in destabilizing RpoS and signifies a "first step" in transitioning from an RpoS-ON state to an RpoS-OFF state. BBD18-mediated post-transcriptional repression of RpoS adds yet another layer of complexity to the sophisticated mechanisms used by *B. burgdorferi* to regulate this critical sigma factor.

## Results

### BBD18 represses RpoS-dependent virulence factors in wild-type *B. burgdorferi*

OspC is an RpoS-dependent virulence factor and the level of RpoS is typically tightly regulated in *B. burgdorferi*, as well as in many other pathogenic bacteria [60], [68]. BBD18 can repress expression of *ospC* in attenuated *B. burgdorferi* strains that demonstrate high *ospC* expression levels under normal in vitro conditions [62]. These strains were developed in vitro by serial passage and selective pressure, resulting in the displacement of most of the *B. burgdorferi* plasmids [69]. The mechanism leading to high level *ospC* expression in these strains, and their precise genetic makeup, was undetermined. Therefore, BBD18-mediated repression of *ospC* was difficult to comprehensively characterize in these strains. To better describe the effect of BBD18 on *ospC* expression, we used a genetically defined wild-type *B. burgdorferi* strain, and analyzed the effect of BBD18 on the production of OspC. We first used allelic exchange to generate an isogenic *rpoS* mutant in our wild-type strain B31-S9, as previously described for the B31-A3 strain [70]. We confirmed the disruption of the *rpoS* locus (B31-S9Δ*rpoS*) and that the plasmid content of the mutant was identical to the parental strain by PCR (data not shown).

**Figure 1:**
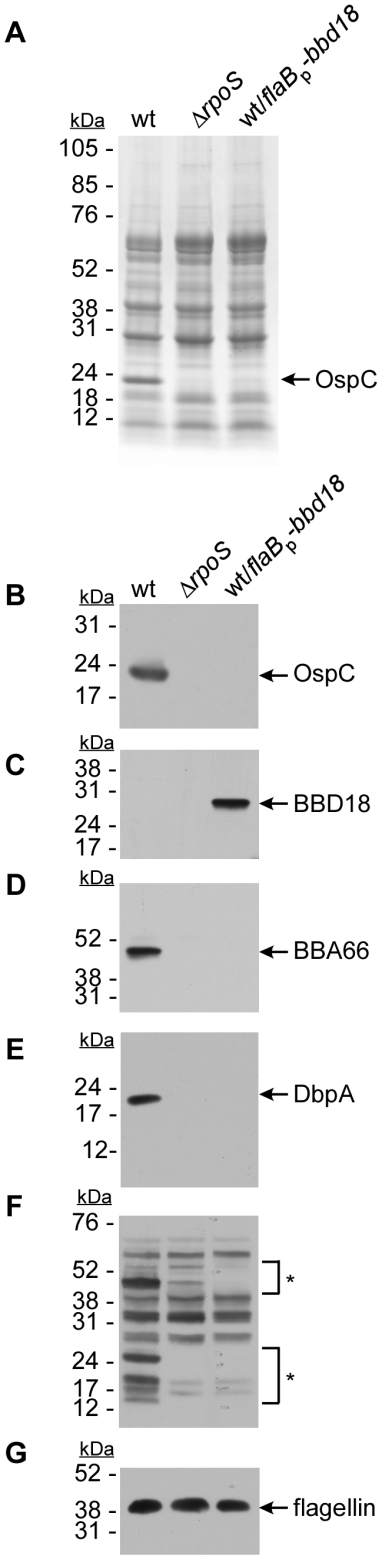
Effect of BBD18 on proteins in the RpoS regulon. *B. burgdorferi* strains B31-S9 (wt), B31-S9Δ*rpoS* (Δ*rpoS*) and B31-S9/pBSV2*-*flaB*p-*bbd18* (wt/*flaB*p-*bbd18*) were grown under *rpoS*-inducing conditions (BSKII medium, pH 6.8). Cell lysates were subjected to SDS-PAGE, Coomassie blue staining (A), and immunoblot analysis (B-G). Membranes were probed for the presence of OspC (B), BBD18 (C), BBA66 (D), or DbpA (E), using protein-specific antibodies or antisera. Pooled sera from mice infected with *B. burgdorferi* by tick bite was used to detect changes in the antigenic protein profile (F). A mouse monoclonal α-flagellin antibody (H9724) was used to detect flagellin as a protein loading control (G). Positions of molecular mass standards are shown on the left in kiloDaltons (kDa).

During normal in vitro growth of *B. burgdorferi*, RpoS levels and RpoS-dependent gene transcription are low [41], [48]. Consistent with these observations [63], we detected low levels of *ospC* transcript or protein under normal in vitro growth conditions (BSKII medium, pH7.6/35°C/5%CO_2_), making it difficult to study the effect of BBD18 on OspC under these conditions (data not shown). However, several in vitro culture conditions have been developed that induce expression of *rpoS*, leading to increased RpoS-dependent gene transcription. These conditions include growing spirochetes at a reduced pH [71], subjecting spirochetes to a temperature shift [15], or growth in increased levels of acetate [61], and are thought to partially mimic the tick-to-mouse transition, the point in the infectious cycle where *rpoS* appears to be maximally expressed in vivo [48], [49]. To induce expression of *rpoS*, and consequently *ospC*, we subjected *B. burgdorferi* strains B31-S9 (wild type), B31-S9Δ*rpoS* (Δ*rpoS*) or B31-S9 containing a shuttle vector constitutively expressing *bbd18* (wild type/*flaB*p*-bbd18*), to growth conditions at a reduced pH (pH6.8). Consistent with previous reports [71], we found that OspC was readily produced by the wild-type strain and that synthesis was RpoS-dependent (Fig. 1, panels A-B) [41], [65]. OspC was undetectable in both the Δ*rpoS* and wild-type/*flaB*p*-bbd18* strains grown under identical conditions (Fig. 1A-B). The absence of OspC in the presence of BBD18 (Fig. 1C) demonstrates that BBD18 expression prevents the production of OspC in *B. burgdorferi* wild-type strains.

A palindromic sequence upstream of the *ospC* promoter plays a role in both the expression and repression of *ospC*, although a specific repressor protein that binds to the putative operator sequence has not been identified [33], [66], [67]. One possible mechanism of BBD18-mediated repression of *ospC* is through the interaction of BBD18 with the *ospC* promoter, either with the inverted repeats (IR) upstream of the *ospC* promoter, or within the region proximal to the transcriptional start site. However, when this potential BBD18-*ospC* promoter interaction was analyzed biochemically using electrophoretic mobility shift assays (EMSA), we found no evidence for a BBD18-*ospC* promoter interaction (Fig. S1). Taken together, these results suggest that BBD18-mediated repression of *ospC* does not involve an interaction of BBD18 with the *ospC* promoter.

In addition to OspC, *B*. *burgdorferi* RpoS-dependent virulence factors BBA66 and DbpA are synthesized early during mammalian infection. To determine if BBD18 production alters any other specific RpoS-dependent virulence factors, we analyzed the production of RpoS-dependent proteins BBA66 and DbpA by immunoblot. While both BBA66 and DbpA were detected in wild type, we were unable to detect these proteins in the Δ*rpoS* strain or when BBD18 was constitutively expressed (Fig. 1, panels D-E). The correlation between the presence of BBD18 and absence of OspC, BBA66, or DbpA suggests that BBD18 expression prevents the production of additional RpoS-dependent virulence factors.

### BBD18 alters the *B. burgdorferi* Antigenic-Protein Profile

Since our analysis was consistent with BBD18 being a global regulator of RpoS-dependent expression, we took advantage of the natural induction of RpoS-dependent proteins that occurs during the infectious cycle [15], [48], [72]. We anticipated that sera collected from mice infected by *B. burgdorferi* could be used to detect global changes in RpoS-dependent protein production and provide a broader assessment of the effect of BBD18 on the *B. burgdorferi* proteome.

We performed Western blots using pooled sera collected from mice infected with *B. burgdorferi* by tick bite, and analyzed cell lysates from wild type, Δ*rpoS*, and wild type/*flaB*p*-bbd18* strains that had been grown under *rpoS*-inducing conditions. In the wild-type strain, we detected several prominent protein bands that were recognized by these sera, demonstrating that proteins normally recognized by the acquired immune response were being produced (Fig. 1F). However, in the Δ*rpoS* and wild type/*flaB*p*-bbd18* strains, many of the immuno-reactive bands were absent or markedly reduced (Fig. 1F, see brackets and asterisks). These proteins were more prominent in wild type, suggesting that their genes are RpoS-dependent. Interestingly, some protein bands appear reduced, although not absent, and likely are not strictly RpoS-dependent, consistent with previous reports demonstrating that some genes in the RpoS regulon are not strictly RpoS-dependent [42], [48]. We do not know the identity of all the proteins recognized by these sera, but they range in molecular mass from ∼12–65 kDa. These data demonstrate that the effect of BBD18 on proteins in the RpoS regulon is not limited to OspC, BBA66, and DbpA. Therefore, BBD18 represses the expression of numerous RpoS-dependent genes, indicating a global regulatory effect imparted by BBD18 on many, if not all, RpoS-dependent proteins.

### BBD18 specifically represses RpoS-dependent gene transcription

**Figure 2:**
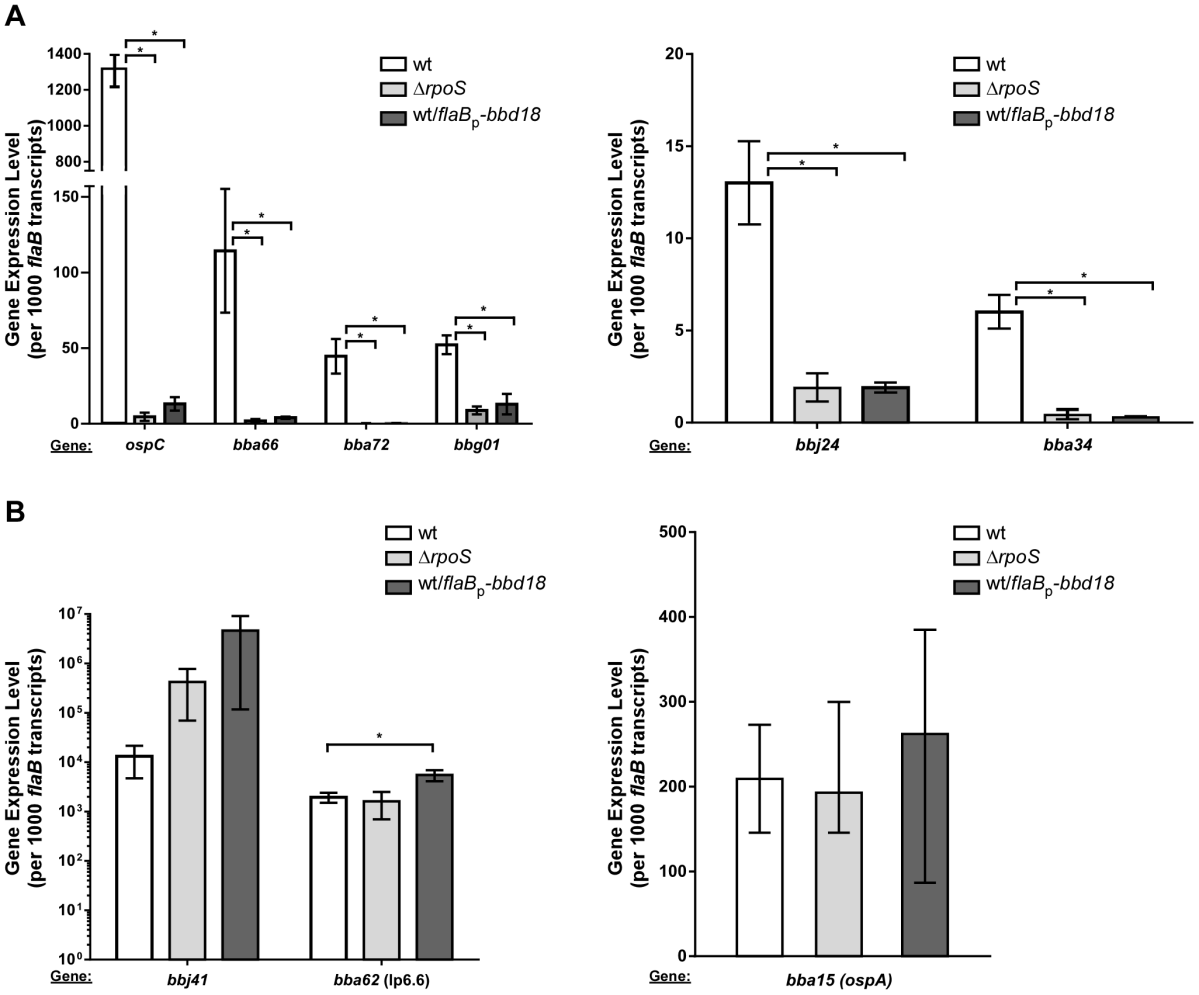
Analysis of RpoS-dependent and RpoS-independent gene transcription. Quantitative reverse transcriptase-PCR (qRT-PCR) analysis of gene expression in strains B31-S9 (wt), B31-S9Δ*rpoS* (Δ*rpoS*) and B31-S9/pBSV2*-*flaB*p-*bbd18* (wt/*flaB*p-*bbd18*), grown under *rpoS-*inducing conditions (BSKII medium, pH6.8). The transcript level of RpoS-dependent genes *ospC*, *bba66*, *bba72*, *bbg01*, *bbj24*, and *bba34* (A) and RpoS-independent genes *bbj41*, *bba62* (*lp6*.6) and *bba15* (*ospA*), (B) are shown as relative units, and normalized to the constitutively expressed *flaB* transcript. Data were analyzed using Student’s unpaired t-test and brackets marked with asterisks represent a statistically significant difference (p<.05).

Having established that BBD18 represses the synthesis or presence of proteins that are RpoS-dependent and normally produced under *rpoS*-inducing conditions, we next sought to determine if BBD18-mediated repression was at the level of transcription. To do so, we determined the transcript level of core genes within the RpoS regulon under *rpoS*-inducing conditions, using quantitative reverse transcriptase PCR (qRT-PCR). We analyzed the transcription of *ospC*, *bba66*, *bba72*, *bbg01, bbj24* and *bba34*, and found that all were readily expressed under *rpoS*-inducing conditions in our wild-type strain (Fig. 2A). Consistent with the RpoS-dependent nature of these genes, transcript levels were all substantially reduced in the Δ*rpoS* strain (Fig. 2A). Under the same *rpoS*-inducing conditions, the wild-type strain constitutively producing BBD18 was unable to induce expression of these genes, and expression levels were similar to those in a Δ*rpoS* strain (Fig. 2A). To determine if BBD18 were acting specifically on RpoS-dependent transcripts or acting as a global repressor of transcription, we analyzed the expression of *rpoS*-independent genes *bbj41, bba62* (lp6.6) and *bba15* (*ospA*). The transcript levels of *bbj41*, *bba62*, and *bba15* are increased in the arthropod vector or during growth conditions mimicking the arthropod vector [73]–[75]. These genes are also subject to RpoS-dependent repression in dialysis membrane chambers in vivo [48], [50]. Our data demonstrate that BBD18 does not repress the transcription of *bbj41, bba62*, or *bba15* (Fig. 2B). These data demonstrate that BBD18-mediated repression is specific for RpoS-dependent transcripts and is not reflective of a global repression of transcription. In fact, *bba62* and *bbj41* show an increase in gene expression in the Δ*rpoS* and wild type/*flaB*p*-bbd18* strains (Fig. 2B). These data indicate that BBD18 represses RpoS-dependent transcripts at the level of transcription, and is specific to RpoS-dependent transcripts.

### BBD18-mediated repression of RpoS is post-transcriptional

**Figure 3:**
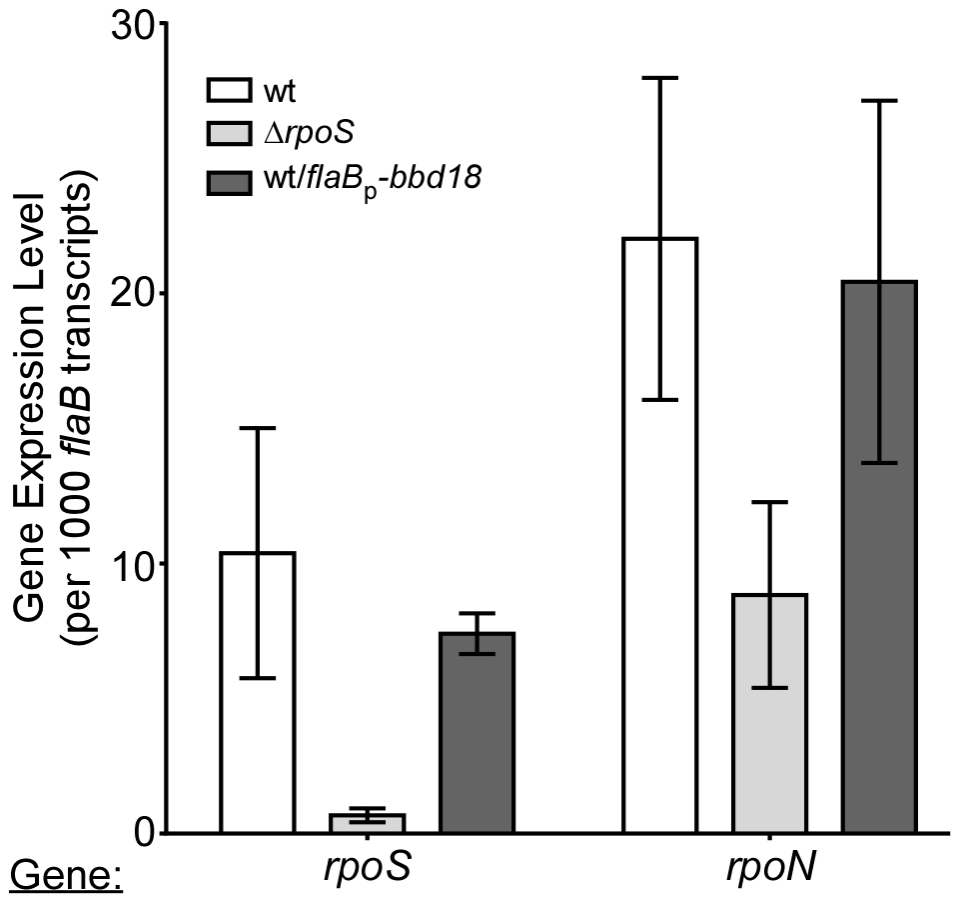
Analysis of the transcript level of alternative sigma factors *rpoN* and *rpoS*. qRT-PCR data displaying the transcript levels of *rpoS* and *rpoN* in strains B31-S9 (wt), B31-S9Δ*rpoS* (Δ*rpoS*) and B31-S9/pBSV2*-*flaB*p-*bbd18* (wt/*flaB*p-*bbd18*) grown under *rpoS*-inducing conditions. Levels of *rpoS* and *rpoN* transcripts are displayed in relative units per 1000 copies of *flaB* transcript. Transcript levels were analyzed using Student’s unpaired t-test and no statistically significant difference was detected (p>.05).

**Figure 4:**
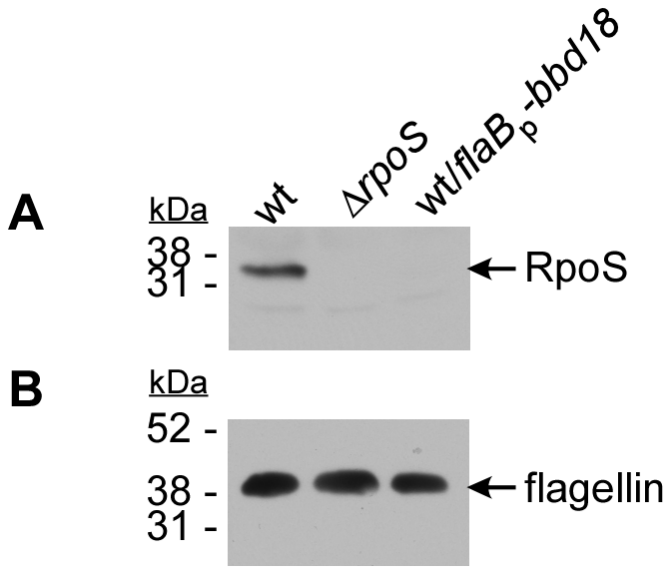
Synthesis of RpoS by wild type but not *bbd18*-expressing *B. burgdorferi* Immunoblot analysis of cell lysates from strains B31-S9 (wt), B31-S9Δ*rpoS* (Δ*rpoS*) and B31-S9/pBSV2*-*flaB*p-*bbd18* (wt/*flaB*p-*bbd18*) grown under *rpoS-*inducing conditions. Cell lysates were analyzed using RpoS antiserum (A) or a mouse monoclonal antibody to flagellin (B) to assess flagellin levels as a protein loading control. Cell lysates and immunoblot for flagellin were the same as those used in Fig. 1. Positions of molecular mass standards are shown on the left in kiloDaltons (kDa).

The gene expression cascade leading to induction of the RpoS regulon is a multistep process [60]. The response regulator protein-2 (Rrp2) is activated and promotes the transcription and subsequent translation of the alternative sigma factor RpoN. RpoN transcribes *rpoS*, and following translation of the *rpoS* transcript, RpoS-dependent gene transcription occurs (Rrp2 -> RpoN -> RpoS -> RpoS-dependent gene transcription)[40], [42]–[44], [51]. Our data establish a role for BBD18 in the specific repression of RpoS-dependent transcripts. However, BBD18 could be exerting its effect on any of the upstream components in the cascade, resulting in the repression of RpoS-dependent gene transcription. To determine where in this regulatory cascade BBD18 exerts its effect, we used qRT-PCR to analyze the transcript levels of the alternative sigma factors *rpoN* and *rpoS*. Since transcription of these genes is low during normal in vitro growth conditions, we analyzed transcripts from *B. burgdorferi* cultured at pH6.8 to induce *rpoS* expression. We found that both *rpoN* and *rpoS* were expressed at similar levels in both wild type and wild type/*flaB*p*-bbd18* strains (Fig. 3). Having detected robust levels of RpoS-dependent transcripts (*ospC*, *bba66*, *bba72*, *bbg01*, *bbj24*, *bba34*) in the wild-type strain and low-to-undetectable levels of RpoS-dependent gene transcripts in the wild type/*flaB*p*-bbd18* strain (compare Fig. 2A and Fig. 3), we found these results surprising. To address this apparent inconsistency, we analyzed cell lysates of spirochetes grown under RpoS-inducing conditions using RpoS-specific antiserum. We found that RpoS was readily detected in the wild-type strain, but not in the Δ*rpoS* or wild type/*flaB*p*-bbd18* strains (Fig. 4). Comparing wild-type to the wild type/*flaB*p*-bbd18* strain, cultured under identical conditions, we found equivalent *rpoS* transcript levels, yet dissimilar protein levels. Taken together, these data demonstrate that BBD18 exerts its regulatory effect on RpoS at a post-transcriptional level.

### BBD18 does not inhibit translation initiation of RpoS

**Figure 5:**
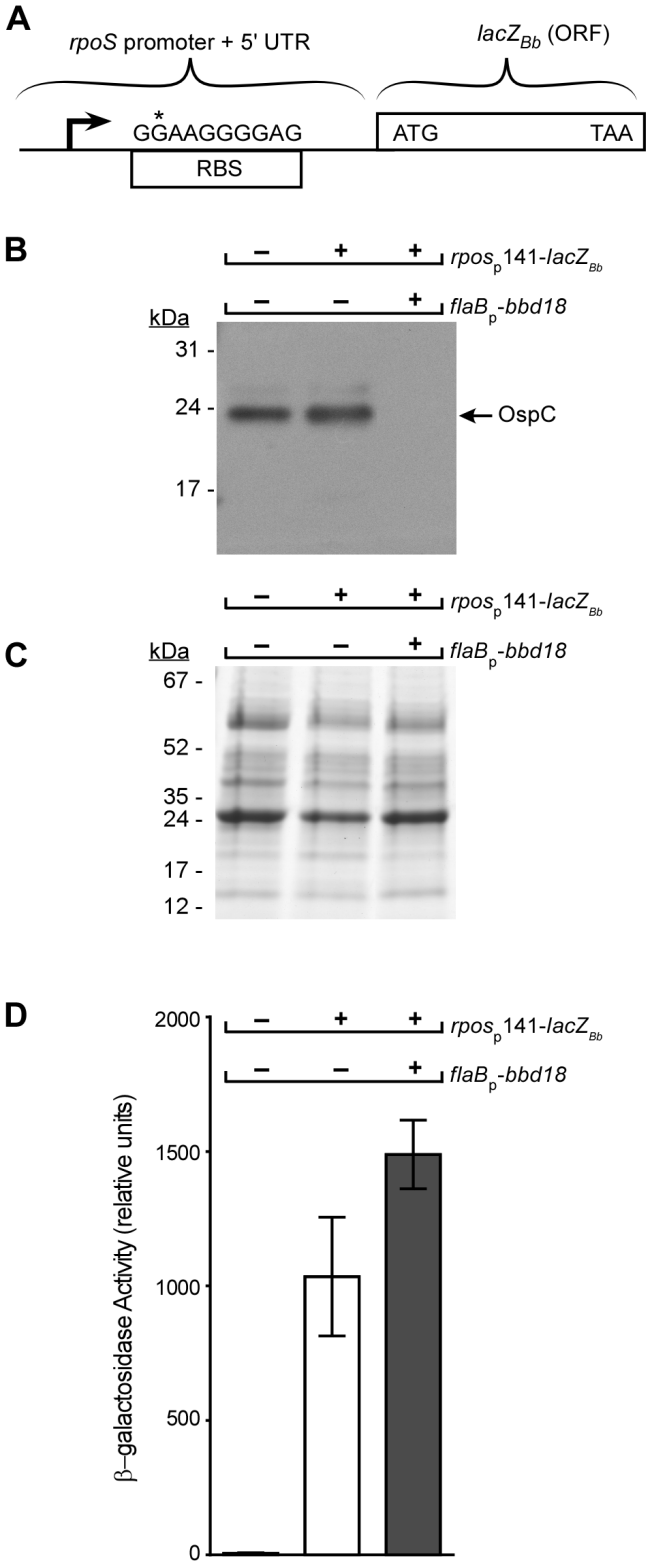
Analysis of BBD18 repression of an *rpoS* promoter *lacZ_Bb_* transcriptional fusion. (A) A schematic diagram of the transcriptional fusion of the *rpoS* promoter and 5’ untranslated region (UTR) fused directly to the *lacZ*_Bb_ open reading frame (ORF). The position of the *rpoS* transcriptional start site [58], [63] is indicated by a filled arrowhead. The Shine-Dalgarno sequence (RBS), and the translational start site of β-galactosidase (*lacZ_Bb_*), indicated by the ATG, are also shown. Regions corresponding to the *rpoS* promoter and 5’ UTR (141bp) or the *lacZ*_Bb_ ORF are indicated with brackets. The "G" in the RBS marked with an asterisk is identified because the reporter construct harbored an A->G mutation relative to the published sequence at that position. Cell lysates from strains B31-S9 (wt), B31-S9/pBSV2G-*rpoS*p*_141_*-*lacZ_Bb_* (wt/*rpoS*p-*lacZ_Bb_*), and B31-S9/pBSV2G- *rpoS*p*_141_*-*lacZ_Bb_*/pBSV28-*flaB*p-*bbd18* (wt/*rpoS*p-*lacZ_Bb_*/*flaB*p-*bbd18*) were grown under *rpoS*-inducing conditions and analyzed with OspC antisera (B) or stained with Coomassie blue (C) to demonstrate equivalent protein loads in each lane. The positions of molecular mass standards are shown on the left in kiloDaltons (kDa). (D) β-galactosidase activity in cell lysates from strains B31-S9 (wt), B31-S9/pBSV2G-*rpoS*p*_141_*-*lacZ_Bb_* (wt/*rpoS*p-*lacZ_Bb_*), and B31-S9/pBSV2G- *rpoS*p*_141_*-*lacZ_Bb_*/pBSV28-*flaB*p-*bbd18* (wt/*rpoS*p-*lacZ_Bb_*/*flaB*p-*bbd18*) grown under *rpoS*-inducing conditions.

A common mechanism of post-transcriptional regulation in bacteria is through inhibition of translation initiation, which can result from the binding of a regulatory factor, or formation of an inhibitory secondary structure that occludes the ribosome binding site and prevents translation [58], [76]–[78]. Because BBD18 exhibits characteristics of a nucleic acid binding protein [62], we hypothesized that BBD18 might interact with the 5' untranslated region (UTR) of the *rpoS* mRNA and inhibit translation. To address this possibility, we constructed a shuttle vector containing a transcriptional fusion of the *rpoS* promoter to the *lacZ_Bb_* reporter gene [79] (Fig. 5A). The transcriptional fusion contains a 141bp region 5’ of the *rpoS* open reading frame (ORF), and includes the transcriptional start site [58], [63], [80] and a Shine-Dalgarno sequence (RBS) [81] (Fig. 5A). The transcriptional fusion between the *rpoS* 5’UTR and the *lacZ_Bb_* gene allows detection of β-galactosidase (β-gal) activity as a measure of translation efficiency from the *rpoS* 5’UTR, and can be used to determine if BBD18-mediated repression of RpoS requires the *rpoS* 5’ UTR.

We first confirmed the activation of the *rpoN*->*rpoS* regulatory cascade in both a wild-type strain and wild type/*flaB*p*-bbd18* harboring the transcriptional fusion (*rpoS*p_141_-*lacZ_Bb_*). Strains were grown under RpoS-inducing conditions (BSKII pH6.8) and cell lysates analyzed for the presence of OspC by Western blot. As expected, we detected OspC in the wild-type strain and the wild type/*rpoS*p_141_-*lacZ_Bb_* strain, but not in the wild type/*rpoS*p_141_-*lacZ_Bb_*/*flaB*p*-bbd18* strain (Fig. 5B). Having established that the RpoS regulon was being transcribed under these conditions, we next measured β-gal activity in these cell lysates. We did not detect any β-gal activity in the wild-type strain lacking the reporter construct (Fig. 5D), whereas we detected significant and equivalent levels of β-gal activity in both strains containing the *rpoS*p_141_-*lacZ_Bb_* transcriptional fusion (Fig. 5D). These data demonstrate that the RpoS regulon is activated under these conditions and, consistent with the qRT-PCR data, suggest that transcription from the *rpoS* promoter is equivalent in both wild type and wild type/*flaB*p*-bbd18* strains. These results suggest that in the wild-type/*flaB*p*-bbd18*/*rpoS*p_141_-*lacZ_Bb_* strain, BBD18 is mediating repression of RpoS and the RpoS regulon, however, it is unable to repress expression of, or translation of, a transcriptional fusion containing the *rpoS* promoter and 5′ UTR (Compare 4B and 4D). Equivalent levels of β–gal activity suggest that BBD18-mediated repression of RpoS is not through inhibition of translation initiation but is likely specific to the RpoS protein. It is important to note that under similar growth conditions, where robust β–gal activity was detected, we were unable to detect RpoS or RpoS-dependent transcripts (Fig 2B and Fig 4). Taken together, these data suggest that BBD18-mediated repression of RpoS is post-transcriptional, specific to the RpoS protein and independent of the *rpoS* promoter and 5'UTR.

## Discussion

To be maintained in nature, *B. burgdorferi* must be transmitted from an infected tick to a susceptible host, establish a persistent infection, and subsequently be re-acquired by feeding ticks. During this alternating tick->mouse->tick infectious cycle, the Lyme disease spirochete remodels its transcriptome in response to specific environmental cues in order to adapt to and survive in these environments. This is accomplished through the use of multiple alternative sigma factors [60]. Exchanging the sigma factor bound to the RNAP holoenzyme allows quick and efficient remodeling of the transcriptome by promoting the transcription of sigma factor-specific genes. Of particular focus in *B. burgdorferi* has been the Rrp2->RpoN->RpoS regulatory cascade, leading to RpoN- and RpoS-dependent gene transcription. This regulatory cascade is turned on early during nymphal tick feeding and controls the expression of virulence factors required for the establishment of mammalian infection [40], [41], [44], [48], [49], [51], [60]. Several environmental cues responsible for turning on this pathway have been identified, and the critical role played by RpoS in virulence factor gene expression in this process has been well established [15], [40], [44], [71], [82]. However, the molecular mechanisms involved in the transition from an RpoS-ON state during nymph feeding (and presumably within a mammalian host) to an RpoS-OFF state in fed larvae and unfed nymphs remains less than well defined. We have now identified a role for a novel factor, BBD18, which likely represents a “first step” in the transition from an RpoS-ON to an RpoS-OFF state in *B. burgdorferi*.

BBD18 was identified originally as a repressor of *ospC* in high passage *B. burgdorferi* strains [62], and initially we focused on the role of BBD18 in the repression of the requisite virulence factor OspC. We have now demonstrated that BBD18 prevents the transcription of RpoS-dependent genes, including *ospC,* in wild-type *B. burgdorferi* indirectly. BBD18 prevents RpoS-dependent gene transcription through its regulatory effect on the alternative sigma factor RpoS, not direct transcriptional repression of genes in the RpoS regulon. Under conditions mimicking the tick-> mammalian transition, where RpoS-dependent gene transcripts are expressed, *bbd18* expression represses transcription of RpoS-dependent genes to levels equivalent to those in an *rpoS* mutant (Fig. 2A). We also detected an increase in the expression of RpoD-dependent transcripts *bba62* and *bbj41* (Fig. 2B) [51]. Since all of the RpoS-dependent transcripts we analyzed were repressed in the presence of constitutive BBD18, we analyzed the transcription level of *rpoS*. Direct analysis by qRT-PCR, and analysis of an *rpoS* transcriptional fusion, demonstrated that the *rpoS* transcript levels, and expression from the *rpoS* promoter, were equivalent to wild type levels when *bbd18* was being constitutively expressed (Fig. 3 and Fig. 5D). However, under similar RpoS-inducing conditions, we were unable to detect RpoS protein when *bbd18* was constitutively expressed (Fig. 4). Moreover, when *rpoS* was induced by a temperature shift from 25**°**C –> 35**°**C, we detected equivalent levels of *rpoS* transcript in wild type and wild type/*flab*p-*bbd18* strains by qRT-PCR, but were unable to detect RpoS protein in strains where *bbd18* was constitutively expressed (data not shown). Additionally, the *rpoS* promoter-*lacZ*_Bb_ transcriptional fusion data (Fig. 5) demonstrate that BBD18-mediated post-transcriptional repression of RpoS is not facilitated through the *rpoS* promoter or 5' UTR, either through direct BBD18 interaction with the RBS, or through sequestration of a translation-promoting factor that interacts with the 5'UTR. Cumulatively, these data suggest that BBD18-mediated repression of RpoS occurs post-transcriptionally, is specific for the RpoS protein, and is likely facilitating, either directly or indirectly, destabilization of RpoS.

In *B. burgdorferi*, RpoS regulation is complex [60]. Protein factors BosR, CsrA, BadR, and HrpA play roles in the regulation of *rpoS* transcription [52]–[54], [56], [83], [84]. Previously, only the small RNA DsrA and RNA-binding protein Hfq were clearly shown to play roles in the post-transcriptional regulation of RpoS in *B. burgdorferi* [58], [59]. DsrA plays a similar role to DsrA in *E. coli,* [85], [86], binding to the 5' UTR of the *rpoS* mRNA, relieving an inhibitory secondary structure, and promoting translation of *rpoS* under certain conditions. One possible mechanism of BBD18-mediated repression of RpoS would be through the sequestration of the *rpoS*-translation-promoting factor DsrA. However, BBD18 was unable to repress translation of β-galactosidase from the transcriptional fusion (*rpoS*_141_p-*lacZ*_Bb_) containing a DsrA binding site (Fig. 5D), making this action through DsrA unlikely. Additionally, following a temperature shift, where DsrA is not active, BBD18-mediated repression of RpoS-dependent transcripts still occurred (data not shown). These results suggest that BBD18-mediated repression is independent of DsrA and the *rpoS* promoter, and specific for the RpoS protein.

A likely mechanism of the RpoS repression described here is through BBD18-mediated targeted degradation of RpoS. Targeted degradation of specific protein factors is characterized in both eukaryotes and prokaryotes as a mechanism to directly reorganize the proteome, as well as modify the transcriptome. Degradation of specific transcriptional activators, repressors, or sigma factors can redirect transcription of specific sets of genes and has been hypothesized as a mechanism for regulating the level of RpoS in *B*. *burgdorferi* [59], [87]–[89]. In *E.coli*, targeted degradation of RpoS has clearly been demonstrated as a robust regulatory mechanism for controlling this alternative sigma factor [45], [90]. This mechanism involves the use of an adaptor protein RssB, which binds to and delivers RpoS to the ClpXP proteasome-like complex, where RpoS is quickly degraded [91]. *B. burgdorferi* harbors homologs of the Clp protease complex, but lacks an RssB homolog. If BBD18 were fulfilling the role of RssB, it may be doing so in a way that is unique to *B. burgdorferi*, as the primary amino acid sequence of BBD18 shares little identity with this factor. However, testing the effect of BBD18 on RpoS repression in a *B*. *burgdorferi* Clp protease mutant has not been possible. There are no available Clp mutants in the transposon mutagenesis library [92], and previous attempts to disrupt these alleles through direct allelic exchange have been unsuccessful (J.A.Carroll, unpublished observations). The Clp protease complex may be essential for cell homeostasis if the loss of Clp proteolytic components leads to unregulated RpoS levels, which are toxic to *B. burgdorferi* [93]. Alternatively, BBD18 might be acting directly on RpoS as a protease, although BBD18 is not homologous to any known protease or contain any proteolytic domains. Preliminary experiments to assess whether the addition of exogenous BBD18 results in enhanced RpoS turnover were inconclusive (data not shown). Whether BBD18 is acting directly as a protease, indirectly as an adaptor, or through some alternative mechanism to destabilize RpoS, remains to be elucidated. Defining the components involved in RpoS destabilization should help determine the molecular mechanisms involved in BBD18-mediated repression of RpoS.

Our data demonstrate that BBD18-mediated repression of the *rpoS* regulon is through regulation of RpoS protein, not at the level of *rpoS* transcription. These data suggest that the role of BBD18 is likely at the point where *B. burgdorferi* needs to transition from having RpoS available for transcription (RpoS-ON), to one where a different alternative sigma factor (presumably σ-70) is required (RpoS-OFF). During the infectious cycle, RpoS must be shut off when spirochetes transition from an infected host to a feeding vector (as well as in unfed nymphs), to allow expression of genes required in the arthropod vector. Targeted degradation of RpoS would allow quick and efficient repression of RpoS activity. RpoS degradation would result in changing the alternative sigma factor bound to RNAP by altering the relative concentration of sigma factors available for RNAP binding. This change would allow *B. burgdorferi* to transition quickly from transcription of mammalian-specific genes like *bba66*, *bba72, bbj24*, *bbg01*, to arthropod-specific genes, like *ospA*, *bbj41* and *bba62*. In fact, we see hints of that possible scenario in this study. Expression of BBD18 led to a decrease in *rpoS*-specific transcripts and a reproducible increase in *rpoD*-dependent transcripts (Fig. 2 A-B). One possible explanation for these data is that a decrease in the level of RpoS makes additional RNAP available for RpoD binding, and thus leads to an increase in RpoD-dependent transcription. Our focus here was on the mode of action of BBD18, and a more comprehensive analysis of the effect of BBD18 would be required to determine if this observation is reproducible and is transcriptome-wide.

**Figure 6:**
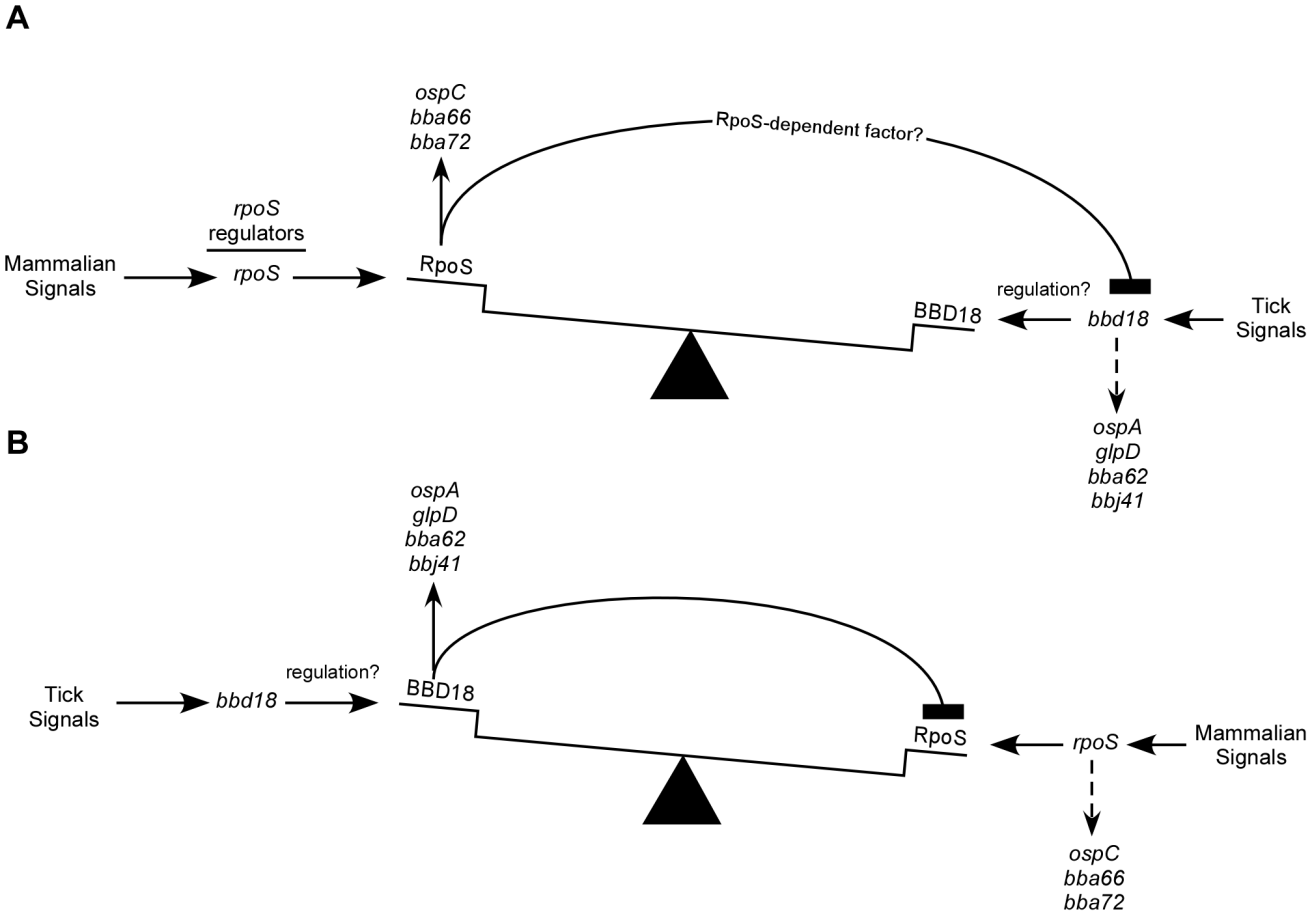
Proposed balance between RpoS and BBD18 in *B. burgdorferi*. (A) In response to mammalian environmental conditions, expression of *rpoS* is induced. Several factors, including BosR, CsrA, Hfq, DsrA and BadR, play roles in regulating *rpoS*. When RpoS protein is available to bind RNAP, it directs the expression of *ospC*, *bba66*, *bba72* and other RpoS-dependent genes. RpoS represses transcription of *bbd18*, *ospA*, *glpD*, *bba62*, *bbj41* and other arthropod-induced genes, possibly through an RpoS-dependent factor[51]. (B) In response to arthropod environmental conditions, *bbd18* is expressed and BBD18 production destabilizes RpoS and represses RpoS-dependent transcription, allowing induction of genes required in the arthropod vector.

Our hypothesis, that BBD18 is required for the host-to-tick transition to repress RpoS-dependent gene transcription, would suggest that *bbd18* is expressed at the beginning of the arthropod phase of the infectious cycle. Analysis of the available microarray and gene expression data for *B. burgdorferi* suggest that this is likely the case. Expression of *bbd18* mimics the expression patterns of genes that are specifically expressed under arthropod-like conditions [73], [74]. Consistent with an in vivo role for BBD18 in the regulation of RpoS at the RpoS ON-> RpoS OFF transition, Tokarz et al. detected *bbd18* transcript in unfed nymphs but not fed nymphs. Also, many of the arthropod-induced genes, including *bbd18*, are transcriptionally repressed in an RpoS-dependent manner in response to mammalian-like environmental signals [48], [50], [51]. Since transcription of *bbd18* appears to be subject to RpoS-mediated repression, and, as our data demonstrate, BBD18 represses RpoS, a delicate balance must exist between the level of BBD18 and the level of RpoS. We have presented a model of how the interplay between RpoS and BBD18 might work (Fig. 6). In response to mammalian signals, *rpoS* transcript levels increase, and subsequent RpoS protein production leads to expression of genes required for the mammalian environment and repression of arthropod-induced genes, including *bbd18* (Fig. 6A). In response to some as-yet unidentified cue upon entering a tick, *bbd18* expression increases, leading to production of BBD18, destabilizing RpoS, allowing de-repression of RpoS-mediated repression and the concomitant expression of arthropod-specific genes (Fig. 6B).

If BBD18 plays a role in the transition from RpoS-ON to RpoS-OFF, it must be regulated appropriately. Inappropriately timed expression of *bbd18* inhibits mammalian infection (Hayes et. al. Submitted). How *bbd18* expression is regulated within ticks and what signals turn on expression of this gene are still undefined. One possibility is an increase in BBD18 activity in response to the phosphorylation state of the protein, similar to the modulation of RssB activity in *E. coli* [94]. Although we do not have evidence for BBD18 phosphorylation, *B. burgdorferi* encodes two histidine kinases whose roles in controlling gene expression have begun to be elucidated [95], [96]. The signals and mechanisms involved in adapting to the arthropod environment and transitioning from mammalian-specific gene expression pattern to the arthropod-specific gene expression pattern are just starting to be examined. Our data demonstrate that expression of *bbd18* likely represents a first step in transitioning from an RpoS-ON to an RpoS-OFF state and as such, its induction might be a harbinger for the induction of arthropod-specific genes.

## Materials and Methods

### *Borrelia burgdorferi* Strains and Growth Conditions

Typically, *B. burgdorferi* strains were cultured in BSKII medium (pH7.6) as previously described [97], [98]. For pH induction of the RpoS regulon, strains were grown in BSKII medium (pH7.6) to late exponential phase (1×10^8^ spirochetes/mL) and transferred to pH-adjusted BSKII medium (pH6.8) at a density of 5×10^7^ spirochetes/mL and allowed to grow to ∼2×10^8^ spirochetes/mL at 35**°**C. Strain B31-S9, termed wild type in this study, is an infectious derivative of B31-A3 that lacks the known restriction modification systems encoded on linear plasmid 56 and linear plasmid 25 that limit introduction of shuttle vector constructs [99]–[101]. Strain B31-S9/pBSV2*-*flaB*p-*bbd18* is a derivative of wild-type B31-S9 strain harboring a shuttle vector constitutively expressing *bbd18* under control of the flagellin (*flaB*) promoter [62]. Strain B31-S9Δ*rpoS* was constructed as previously described [70] and disruption of the *rpoS* locus and plasmid content verified by PCR (primers 3 & 4, Table S1). Where appropriate, kanamycin, streptomycin and gentamicin were used at 200 ug/mL, 50 ug/mL or 40 ug/mL, respectively.

### SDS-PAGE and Immunoblots

For analysis of cell lysates by SDS-PAGE and Western blot, equivalent numbers of spirochetes were harvested by centrifugation based on enumerating spirochetes in a Petroff-Hausser chamber. Spirochetes were washed twice in HN buffer (50 mM Hepes (pH 7.5), 50 mM NaCl), and resuspended in equal volumes of Laemmli gel loading buffer for protein analysis, or mixed with TRIzol reagent (Life Technologies, Carlsbad CA) and stored at -80°C for RNA extraction. Protein samples were resolved by SDS-PAGE on 4–15% gradient gels (Bio-Rad, Hercules CA) and either stained with Coomassie blue or transferred to a nitrocellulose membrane for immunoblotting. For detection of OspC, DbpA, RpoS, FlaB, BBD18, and proteins detected with infected-mouse sera, membranes were blocked for 1 hr in a 4% nonfat milk solution (Lab Scientific, Livingston, NJ) prepared in Tris-buffered saline containing 0.1% Tween 20 (TBST-20), with rocking. Membranes were then transferred to a 1% solution of nonfat milk in TBST-20 containing one of the following: rabbit α-OspC polyclonal antiserum (1∶1000) [32], rabbit α-RpoS polyclonal antiserum (UGA-17, 1∶500), α-DbpA purified antibody (Rockland Immunochemicals, Gilbertsville, PA, 401-B98, 1∶500), mouse monoclonal α-FlaB antibody (H9724, 1∶500) [102], rabbit α-BBD18 polyclonal antiserum (1∶500), generated, as described [103], using purified recombinant BBD18 protein (see below), or pooled infected-mouse sera (pooled from several mice infected with wild-type B31-A3 spirochetes by tick transmission, 1∶200). Immunoblots were then incubated in TBST-20 with the appropriate peroxidase-conjugated secondary antibody for 30 min and developed using SuperSignal West Pico chemiluminescent substrate kit (Thermo Scientific, Rockford, IL) and X-ray film (Lab Scientific Inc., Livingston, NJ). Detection of BBA66 was performed as previously described [46].

### Quantitative reverse transcriptase PCR (qRT-PCR)

Total RNA was isolated from *B. burgdorferi* strains using TRIzol (Life Technologies, Carlsbad, CA) reagent following the manufacturer's instructions. Subsequently, 5 ug of RNA from each strain was treated with DNAse for 1 hr at 37**°**C using TurboDNase (Life Technologies) and purified following the manufacturer’s protocol. Following purification, 1µg of RNA was used to generate cDNA using High Capacity cDNA Reverse Transcriptase kit (Life Technologies), following the manufacturer's protocol. Quantitative PCR (qPCR) reactions were performed using TaqMan Universal PCR Mastermix (Life Technologies) with gene specific primer and probe sets (primer 2 nM each, probe 5 nM) (Integrated DNA Technologies, Coralville, IA) (Table S1). Experiments were performed in biological and technical triplicate and analyzed on an ABI 7900 using Sequence Detection Software (SDS 2.4), or a Viia7 using the Viia 7 software package (Life Technologies). The mean and standard deviations were determined using PRISM software (PRISM). qPCR reactions performed on cDNA samples generated in the absence of reverse transcriptase were similar to no-template control reactions.

### β-Galactosidase Activity Assays

A 141bp fragment upstream of *rpoS*, containing the *rpoS* promoter sequence, was amplified by PCR using primers 7 & 8 (Table S1) and cloned into *SalI* and *BspHI* sites immediately upstream of the *lacZ_Bb_* gene on pBHlacZ_Bb_* [62], creating pBSV2G-*rpoS*p_141_-*lacZ_Bb_*. Construction of pBSV28-*flaB*p-*bbd18* shuttle vector, containing the flagellin (*flaB*) promoter driving expression of *bbd18* on pBSV28, is described elsewhere (Hayes et. al. Submitted). The relevant plasmid sequences were verified by Sanger sequencing and the pBSV2G-*rpoS*p_141_-*lacZ_Bb_* plasmid contained an A->G mutation in the RBS relative to the published sequence. The pBSV2G-*rpoS*p_141_-*lacZ_Bb_* shuttle vector was transformed into strain B31-S9 and transformants were confirmed by PCR using primers 1 and 2 (Table S1). B31-S9/pBSV2G-*rpoS*p_141_-*lacZ_Bb_* was then transformed with pBSV28-*flaB*p-*bbd18* generating B31-S9/pBSV28-*flaB*p*-bbd18/*pBSV2G-*rpoS*p_141_-*lacZ_Bb_*. For analysis of β-galactosidase activity, strains B31-S9, B31-S9/pBSV2G-*rpoS*p_141_- *lacZ_Bb_*, B31-S9/pBSV28-*flaB*p*-bbd18/*pBSV2G-*rpoS*p_141_-*lacZ_Bb_* were grown in BSKII medium (pH7.6) to late exponential phase, transferred to BSKII medium (pH6.8) at a density of 5×10^7^ spirochetes/mL, and allowed to grow to ∼2×10^8^ spirochetes/mL at 35**°**C. 1 ml cultures containing equivalent numbers of spirochetes were harvested by centrifugation and washed 3 times with HN buffer. β -galactosidase activity was determined using the Galacto-Light Plus Chemiluminescent reporter gene assay system (Life Technologies) following the manufacturer's protocol for microplate detection. Briefly, spirochetes were lysed in Lysis Solution, cell debris was cleared by centrifugation and, after a 20 min incubation with the chemiluminescent substrate, β-galactosidase activity was measured in a microtiter plate using a BioTek Synergy-2 plate reader (BioTek, Winooski, VT). Each sample was analyzed in triplicate following the manufacturer's instructions. To ensure equivalent sample loads between wells, sample volumes were normalized based on their absorbance reading at 260nm. Luminescence readings were measured in relative units and the mean and standard deviation from three independent experiments were determined using PRISM software. To check induction of the RpoS regulon, a portion of the lysate used in the β-galactosidase assay was analyzed by immunoblot for the presence of OspC, as described above.

### Expression and Purification of BBD18

The *bbd18* open reading frame was amplified by PCR from low passage wild-type B31-A3 genomic DNA using primers 5 & 6 (Table S1). The resulting product was digested with NdeI and XhoI (New England Biolabs, Ipswich, MA) and purified using a PCR purification kit (Qiagen, Valencia, CA). The purified product was ligated into similarly-digested pET28 (EMD Millipore, Billerica, Massachusetts) in frame with an N-terminal six-histidine tag, creating pET28-6XHis-*bbd18*. The relevant portion of this plasmid was confirmed by Sanger sequencing and transformed into BL21-CodonPlus (DE3)-RIPL *E. coli* (Agilent Technologies, Santa Clara,CA) for protein expression. Typically, BL21-CodonPlus(DE3)-RIPL/pET28-6XHis-*bbd18* were grown in 1 to 4L of LB at 37**°**C to an O.D._600_ of 0.5 with shaking. Isopropyl-β-D-1 thiogalactopyranoside (IPTG) was added to a final concentration of 1 mM for induction of expression. Expression continued for 3 hr and cultures were harvested by centrifugation and stored at –80**°**C. Cell pellets were resuspended in 30 mL of lysis buffer (1 M NH_4_Cl, 150 mM KCl, 50 mM Hepes (pH 7.5), 2 mM β-mercaptoethanol, 10 mM imidazole, 5 mM MgCl_2_), disrupted by sonication, and cell debris was cleared by centrifugation. The soluble fraction was combined with 1 mL of pre-equilibrated Ni-NTA slurry and allowed to incubate for 1hr at 4**°**C with rocking. Following incubation, the slurry was applied to a chromatography column and washed with 100 bed volumes of lysis buffer. Proteins were eluted from the column in 5 mL fractions using elution buffer (300 mM KCl, 50 mM Hepes (pH 7.5), 2 mM β-mercaptoethanol, 250 mM Imidazole, 5 mM MgCl_2_). Elution fractions were analyzed by SDS-PAGE and Coomassie blue staining and the identity of BBD18 was confirmed by mass spectroscopy. Elution fractions containing BBD18 were combined in an Amicon Ultra centrifugal unit (EMD Millapore, Billerica, MA) and subjected to buffer exchange and protein concentration into a buffer lacking imidazole (300 mM KCl, 50 mM Hepes (pH 7.5), 2 mM β-mercaptoethanol, 5 mM MgCl_2_). BBD18 antiserum was generated as previously described [103].

### Gel Mobility Shift Assays

Gel mobility shift assays were performed as in [104]. Briefly, pPCR8-*ospC*p-5F containing the *ospC* promoter and upstream inverted repeats was digested with Fok1 (New England Biolabs) and purified using a PCR purification kit (Qiagen). Restriction digested pPCR8-*ospC*p-5F (400 ng) was incubated with 6 μM to 150 μM of purified BBD18 at 25°C for 15 min in binding buffer (10 mM Hepes pH 7.5, 2 mM β-mercaptoethanol, 1 mM MgCl_2_, 1 mg/mL BSA, 0.01% NP40). Samples were resolved on 5% nondenaturing polyacrylamide gels in cold 0.5X TBE and visualized by staining gels with a 1X solution of Gel Red Nucleic Acid Stain (Biotium, Hayward, CA).

## Supporting Information

Figure S1 **Analysis of a potential BBD18-*ospC* promoter interaction.** Recombinant BBD18 was purified by affinity chromatography and analyzed by SDS-PAGE and Coomassie blue staining (A). Electrophoretic mobility shift assay of restriction digested pPCR8-*ospC*p-5F, containing the *ospC* promoter and upstream inverted repeats, was incubated with purified recombinant BBD18 and resolved on a 5% polyacrylamide gel (B). The arrow indicating unbound DNA is directed at the specific restriction fragment containing the *ospC* promoter and upstream inverted repeats.
(TIF)

Table S1 **Oligonucleotide primers and probes used in this study.**
(DOCX)

## References

[1] Burgdorfer W, Barbour AG, Hayes SF, Benach JL, Grunwaldt E, et al (1982) Lyme disease - a tick-borne spirochetosis? Science 216: 1317–1319. 7043737 10.1126/science.7043737

[2] Steere AC, Grodzicki RL, Kornblatt AN, Craft JE, Barbour AG, et al (1983) The spirochetal etiology of Lyme disease. N Engl J Med 308: 733–740. 6828118 10.1056/NEJM198303313081301

[3] Steere AC (1989) Lyme disease. N Engl J Med 321: 586–596. 2668764 10.1056/NEJM198908313210906

[4] Lane RS, Piesman J, Burgdorfer W (1991) Lyme borreliosis: relation of its causative agent to its vectors and hosts in North America and Europe. Annu Rev Entomol 36: 587–609. 2006870 10.1146/annurev.en.36.010191.003103

[5] Barbour AG, Hayes SF (1986) Biology of *Borrelia* species. Microbiol Rev 50: 381–400. 3540570 10.1128/mr.50.4.381-400.1986 PMC373079

[6] Piesman J, Oliver JR, Sinsky RJ (1990) Growth kinetics of the Lyme disease spirochete (*Borrelia burgdorferi*) in vector ticks (*Ixodes dammini*). Am J Trop Med Hyg 42: 352–357. 2331043 10.4269/ajtmh.1990.42.352

[7] Magnarelli LA, Anderson JF (1988) Ticks and biting insects infected with the etiologic agent of Lyme disease, *Borrelia burgdorferi* . J Clin Microbiol 26: 1482–1486. 3170711 10.1128/jcm.26.8.1482-1486.1988 PMC266646

[8] Boylan JA, Lawrence KA, Downey JS, Gherardini FC (2008) *Borrelia burgdorferi* membranes are the primary targets of reactive oxygen species. Mol Microbiol 68: 786–799. 18373524 10.1111/j.1365-2958.2008.06204.x PMC2327290

[9] Bourret TJ, Boylan JA, Lawrence KA, Gherardini FC (2011) Nitrosative damage to free and zinc-bound cysteine thiols underlies nitric oxide toxicity in wild-type *Borrelia burgdorferi* . Mol Microbiol 81: 259–273. 21564333 10.1111/j.1365-2958.2011.07691.x PMC3147059

[10] de Silva AM, Fikrig E, Hodzic E, Kantor FS, Telford SR 3rd, et al (1998) Immune evasion by tickborne and host-adapted *Borrelia burgdorferi* . J Infect Dis 177: 395–400. 9466527 10.1086/514200

[11] Ma Y, Seiler KP, Tai KF, Yang L, Woods M, et al (1994) Outer surface lipoproteins of *Borrelia burgdorferi* stimulate nitric oxide production by the cytokine-inducible pathway. Infect Immun 62: 3663–3671. 7520417 10.1128/iai.62.9.3663-3671.1994 PMC303016

[12] Xu Q, McShan K, Liang FT (2008) Essential protective role attributed to the surface lipoproteins of *Borrelia burgdorferi* against innate defenses. Mol Microbiol 69: 15–29. 18452586 10.1111/j.1365-2958.2008.06264.x PMC2574894

[13] Wang G, Ma Y, Buyuk A, McClain S, Weis JJ, et al (2004) Impaired host defense to infection and Toll-like receptor 2-independent killing of *Borrelia burgdorferi* clinical isolates in TLR2-deficient C3H/HeJ mice. FEMS Microbiol Lett 231: 219–225. 14987768 10.1016/S0378-1097(03)00960-1

[14] Montgomery RR, Lusitani D, de Boisfleury Chevance A, Malawista SE (2002) Human phagocytic cells in the early innate immune response to *Borrelia burgdorferi* . J Infect Dis 185: 1773–1779. 12085324 10.1086/340826

[15] Schwan TG, Piesman J, Golde WT, Dolan MC, Rosa PA (1995) Induction of an outer surface protein on *Borrelia burgdorferi* during tick feeding. Proc Natl Acad Sci USA 92: 2909–2913. 7708747 10.1073/pnas.92.7.2909 PMC42328

[16] Schwan TG, Piesman J (2000) Temporal changes in outer surface proteins A and C of the Lyme disease-associated spirochete, *Borrelia burgdorferi*, during the chain of infection in ticks and mice. J Clin Microbiol 39: 382–388. 10.1128/jcm.38.1.382-388.2000 PMC88728 10618120

[17] Gilmore RD, Howison RR, Schmit VL, Nowalk AJ, Clifton DR, et al (2007) Temporal expression analysis of *Borrelia burgdorferi* paralogous gene family 54 genes BBA64, BBA65 and BBA66 during persistent infection in mice. Infect Immun 75: 2753–2764. 17371862 10.1128/IAI.00037-07 PMC1932849

[18] Blevins JS, Hagman KE, Norgard MV (2008) Assessment of decorin-binding protein A to the infectivity of *Borrelia burgdorferi* in the murine models of needle and tick infection. BMC Microbiol 8: 82. 18507835 10.1186/1471-2180-8-82 PMC2430964

[19] Fischer JR, Parveen N, Magoun L, Leong JM (2003) Decorin-binding proteins A and B confer distinct mammalian cell type-specific attachment by *Borrelia burgdorferi*, the Lyme disease spirochete. Proc Natl Acad Sci U S A 100: 7307–7312. 12773620 10.1073/pnas.1231043100 PMC165871

[20] Yang XF, Pal U, Alani SM, Fikrig E, Norgard MV (2004) Essential role for OspA/B in the life cycle of the Lyme disease spirochete. J Exp Med 199: 641–648. 14981112 10.1084/jem.20031960 PMC2213294

[21] Grimm D, Tilly K, Byram R, Stewart PE, Krum JG, et al (2004) Outer-surface protein C of the Lyme disease spirochete: A protein induced in ticks for infection of mammals. Proc Natl Acad Sci USA 101: 3142–3147. 14970347 10.1073/pnas.0306845101 PMC365757

[22] Battisti JM, Bono JL, Rosa PA, Schrumpf ME, Schwan TG, et al (2008) Outer surface protein A protects Lyme disease spirochetes from acquired host immunity in the tick vector. Infect Immun 76: 5228–5237. 18779341 10.1128/IAI.00410-08 PMC2573341

[23] de Silva AM, Fish D, Burkot TR, Zhang Y, Fikrig E (1997) OspA antibodies inhibit the acquisition of *Borrelia burgdorferi* by *Ixodes* ticks. Infect Immun 65: 3146–3150. 9234767 10.1128/iai.65.8.3146-3150.1997 PMC175444

[24] Ohnishi J, Piesman J, de Silva AM (2001) Antigenic and genetic heterogeneity of *Borrelia burgdorferi* populations transmitted by ticks. Proc Natl Acad Sci USA 98: 670–675. 11209063 10.1073/pnas.98.2.670 PMC14646

[25] Pappas CJ, Iyer R, Petzke MM, Caimano MJ, Radolf JD, et al (2011) *Borrelia burgdorferi* requires glycerol for maximum fitness during the tick phase of the enzootic cycle. PLoS Pathog 7: e1002102. 21750672 10.1371/journal.ppat.1002102 PMC3131272

[26] He M, Oman T, Xu H, Blevins J, Norgard MV, et al (2008) Abrogation of *ospAB* constitutively activates the Rrp2-RpoN-RpoS pathway (sigmaN-sigmaS cascade) in *Borrelia burgdorferi* . Mol Microbiol 70: 1453–1464. 19019147 10.1111/j.1365-2958.2008.06491.x PMC2792205

[27] Xu Q, Seemanaplli SV, McShan K, Liang FT (2007) Increasing the interaction of *Borrelia burgdorferi* with decorin significantly reduces the 50 percent infectious dose and severely impairs dissemination. Infect Immun 75: 4272–4281. 17562764 10.1128/IAI.00560-07 PMC1951149

[28] Patton TG, Brandt KS, Nolder C, Clifton DR, Carroll JA, et al (2013) *Borrelia burgdorferi bba66* gene inactivation results in attenuated mouse infection by tick transmission. Infect Immun 81: 2488–2498. 23630963 10.1128/IAI.00140-13 PMC3697616

[29] Xu Q, Seemanapalli SV, McShan K, Liang FT (2006) Constitutive expression of outer surface protein C diminishes the ability of *Borrelia burgdorferi* to evade specific humoral immunity. Infect Immun 74: 5177–5184. 16926410 10.1128/IAI.00713-06 PMC1594837

[30] Shi Y, Xu Q, McShan K, Liang FT (2008) Both decorin-binding proteins A and B are critical for the overall virulence of Borrelia burgdorferi. Infect Immun 76: 1239–1246. 18195034 10.1128/IAI.00897-07 PMC2258843

[31] Weening EH, Parveen N, Trzeciakowski JP, Leong JM, Hook M, et al (2008) Borrelia burgdorferi lacking DbpBA exhibits an early survival defect during experimental infection. Infect Immun 76: 5694–5705. 18809667 10.1128/IAI.00690-08 PMC2583571

[32] Tilly K, Bestor A, Jewett MW, Rosa P (2007) Rapid clearance of Lyme disease spirochetes lacking OspC from skin. Infect Immun 75: 1517–1519. 17158906 10.1128/IAI.01725-06 PMC1828573

[33] Xu Q, McShan K, Liang FT (2007) Identification of an *ospC* operator critical for immune evasion of *Borrelia burgdorferi* . Mol Microbiol 64: 220–236. 17376084 10.1111/j.1365-2958.2007.05636.x

[34] Xu Q, McShan K, Liang FT (2008) Modification of *Borrelia burgdorferi* to overproduce OspA or VlsE alters its infectious behaviour. Microbiology 154: 3420–3429. 18957595 10.1099/mic.0.2008/019737-0

[35] Osterberg S, del Peso-Santos T, Shingler V (2011) Regulation of alternative sigma factor use. Annu Rev Microbiol 65: 37–55. 21639785 10.1146/annurev.micro.112408.134219

[36] Manganelli R, Provvedi R, Rodrigue S, Beaucher J, Gaudreau L, et al (2004) Sigma factors and global gene regulation in Mycobacterium tuberculosis. J Bacteriol 186: 895–902. 14761983 10.1128/JB.186.4.895-902.2004 PMC344228

[37] Dong T, Coombes BK, Schellhorn HE (2009) Role of RpoS in the virulence of Citrobacter rodentium. Infect Immun 77: 501–507. 18981255 10.1128/IAI.00850-08 PMC2612282

[38] Fraser CM, Casjens S, Huang WM, Sutton GG, Clayton R, et al (1997) Genomic sequence of a Lyme disease spirochaete, *Borrelia burgdorferi* . Nature 390: 580–586. 9403685 10.1038/37551

[39] Casjens SR, Mongodin EF, Qiu WG, Luft BJ, Schutzer SE, et al (2012) Genome Stability of Lyme Disease Spirochetes: Comparative Genomics of *Borrelia burgdorferi* Plasmids. PLoS One 7: e33280. 22432010 10.1371/journal.pone.0033280 PMC3303823

[40] Dunham-Ems SM, Caimano MJ, Eggers CH, Radolf JD (2012) *Borrelia burgdorferi* requires the alternative sigma factor RpoS for dissemination within the vector during tick-to-mammal transmission. PLoS Pathog 8: e1002532. 22359504 10.1371/journal.ppat.1002532 PMC3280991

[41] Caimano MJ, Eggers CH, Hazlett KR, Radolf JD (2004) RpoS is not central to the general stress response in *Borrelia burgdorferi* but does control expression of one or more essential virulence determinants. Infect Immun 72: 6433–6445. 15501774 10.1128/IAI.72.11.6433-6445.2004 PMC523033

[42] Fisher MA, Grimm D, Henion AK, Elias AF, Stewart PE, et al (2005) *Borrelia burgdorferi* sigma54 is required for mammalian infection and vector transmission but not for tick colonization. Proc Natl Acad Sci USA 102: 5162–5167. 15743918 10.1073/pnas.0408536102 PMC555983

[43] Ouyang Z, Blevins JS, Norgard MV (2008) Transcriptional interplay among the regulators Rrp2, RpoN and RpoS in *Borrelia burgdorferi* . Microbiology 154: 2641–2658. 18757798 10.1099/mic.0.2008/019992-0

[44] Hübner A, Wang X, Nolen DM, Popova TG, Cabello FC, et al (2001) Expression of *Borrelia burgdorferi* OspC and DbpA is controlled by a RpoN-RpoS regulatory pathway. Proc Natl Acad Sci USA 98: 12724–12729. 11675503 10.1073/pnas.231442498 PMC60121

[45] Battesti A, Majdalani N, Gottesman S (2011) The RpoS-mediated general stress response in *Escherichia coli* . Annu Rev Microbiol 65: 189–213. 21639793 10.1146/annurev-micro-090110-102946 PMC7356644

[46] Clifton DR, Nolder CL, Hughes JL, Nowalk AJ, Carroll JA (2006) Regulation and expression of *bba66* encoding an immunogenic infection-associated lipoprotein in *Borrelia burgdorferi* . Mol Microbiol 61: 243–258. 16824109 10.1111/j.1365-2958.2006.05224.x

[47] Tilly K, Krum JG, Bestor A, Jewett MW, Grimm D, et al (2006) *Borrelia burgdorferi* OspC protein required exclusively in a crucial early stage of mammalian infection. Infect Immun 74: 3554–3564. 16714588 10.1128/IAI.01950-05 PMC1479285

[48] Caimano MJ, Iyer R, Eggers CH, Gonzalez C, Morton EA, et al (2007) Analysis of the RpoS regulon in *Borrelia burgdorferi* in response to mammalian host signals provides insight into RpoS function during the enzootic cycle. Mol Microbiol 65: 1193–1217. 17645733 10.1111/j.1365-2958.2007.05860.x PMC2967192

[49] Ouyang Z, Narasimhan S, Neelakanta G, Kumar M, Pal U, et al (2012) Activation of the RpoN-RpoS regulatory pathway during the enzootic life cycle of *Borrelia burgdorferi* . BMC Microbiol 12: 44. 22443136 10.1186/1471-2180-12-44 PMC3320556

[50] Mulay VB, Caimano MJ, Iyer R, Dunham-Ems S, Liveris D, et al (2009) *Borrelia burgdorferi bba74* is expressed exclusively during tick feeding and is regulated by both arthropod- and mammalian host-specific signals. J Bacteriol 191: 2783–2794. 19218390 10.1128/JB.01802-08 PMC2668432

[51] Caimano MJ, Eggers CH, Gonzalez CA, Radolf JD (2005) Alternate sigma factor RpoS is required for the in vivo-specific repression of *Borrelia burgdorferi* plasmid lp54-borne *ospA* and *lp6.6* genes. J Bacteriol 187: 7845–7852. 16267308 10.1128/JB.187.22.7845-7852.2005 PMC1280317

[52] Ouyang Z, Kumar M, Kariu T, Haq S, Goldberg M, et al (2009) BosR (BB0647) governs virulence expression in *Borrelia burgdorferi* . Mol Microbiol 74: 1331–1343. 19889086 10.1111/j.1365-2958.2009.06945.x PMC2831293

[53] Karna SL, Sanjuan E, Esteve-Gassent MD, Miller CL, Maruskova M, et al (2011) CsrA modulates levels of lipoproteins and key regulators of gene expression critical for pathogenic mechanisms of *Borrelia burgdorferi* . Infect Immun 79: 732–744. 21078860 10.1128/IAI.00882-10 PMC3028860

[54] Miller CL, Karna SL, Seshu J (2013) *Borrelia* host adaptation Regulator (BadR) regulates *rpoS* to modulate host adaptation and virulence factors in *Borrelia burgdorferi* . Mol Microbiol 88: 105–124. 23387366 10.1111/mmi.12171 PMC4828661

[55] Van Laar TA, Lin YH, Miller CL, Karna SL, Chambers JP, et al (2012) Effect of levels of acetate on the mevalonate pathway of *Borrelia burgdorferi* . PLoS One 7: e38171. 22675445 10.1371/journal.pone.0038171 PMC3364977

[56] Boylan JA, Posey JE, Gherardini FC (2003) *Borrelia* oxidative stress response regulator, BosR: a distinctive Zn-dependent transcriptional activator. Proc Natl Acad Sci USA 100: 11684–11689. 12975527 10.1073/pnas.2032956100 PMC208818

[57] Seshu J, Boylan JA, Hyde JA, Swingle KL, Gherardini FC, et al (2004) A conservative amino acid change alters the function of BosR, the redox regulator of *Borrelia burgdorferi* . Mol Microbiol 54: 1352–1363. 15554974 10.1111/j.1365-2958.2004.04352.x

[58] Lybecker MC, Samuels DS (2007) Temperature-induced regulation of RpoS by a small RNA in *Borrelia burgdorferi* . Mol Microbiol 64: 1075–1089. 17501929 10.1111/j.1365-2958.2007.05716.x

[59] Lybecker MC, Abel CA, Feig AL, Samuels DS (2010) Identification and function of the RNA chaperone Hfq in the Lyme disease spirochete *Borrelia burgdorferi* . Mol Microbiol 78: 622–635. 20815822 10.1111/j.1365-2958.2010.07374.x PMC2963666

[60] Samuels DS (2011) Gene regulation in *Borrelia burgdorferi* . Annu Rev Microbiol 65: 479–499. 21801026 10.1146/annurev.micro.112408.134040

[61] Xu H, Caimano MJ, Lin T, He M, Radolf JD, et al (2010) Role of acetyl-phosphate in activation of the Rrp2-RpoN-RpoS pathway in *Borrelia burgdorferi* . PLoS Pathog 6: e1001104. 20862323 10.1371/journal.ppat.1001104 PMC2940757

[62] Sarkar A, Hayes BM, Dulebohn DP, Rosa PA (2011) Regulation of the virulence determinant OspC by *bbd18* on linear plasmid lp17 of *Borrelia burgdorferi* . J Bacteriol 193: 5365–5373. 21784941 10.1128/JB.01496-10 PMC3187453

[63] Burtnick MN, Downey JS, Brett PJ, Boylan JA, Frye JG, et al (2007) Insights into the complex regulation of *rpoS* in *Borrelia burgdorferi* . Mol Microbiol 65: 277–293. 17590233 10.1111/j.1365-2958.2007.05813.x PMC1976401

[64] Yang XF, Alani SM, Norgard MV (2003) The response regulator Rrp2 is essential for the expression of major membrane lipoproteins in *Borrelia burgdorferi* . Proc Natl Acad Sci USA 100: 11001–11006. 12949258 10.1073/pnas.1834315100 PMC196916

[65] Yang XF, Lybecker MC, Pal U, Alani SM, Blevins J, et al (2005) Analysis of the *ospC* regulatory element controlled by the RpoN-RpoS regulatory pathway in *Borrelia burgdorferi* . J Bacteriol 187: 4822–4829. 15995197 10.1128/JB.187.14.4822-4829.2005 PMC1169512

[66] Xu Q, McShan K, Liang FT (2008) Verification and dissection of the *ospC* operator by using *flaB* promoter as a reporter in *Borrelia burgdorferi* . Microb Pathog 45: 70–78. 18479884 10.1016/j.micpath.2008.03.002 PMC2497006

[67] Drecktrah D, Hall LS, Hoon-Hanks LL, Samuels DS (2013) An inverted repeat in the *ospC* operator is required for induction in *Borrelia burgdorferi* . PLoS One 8: e68799. 23844242 10.1371/journal.pone.0068799 PMC3700930

[68] Dong T, Schellhorn HE (2010) Role of RpoS in virulence of pathogens. Infect Immun 78: 887–897. 19948835 10.1128/IAI.00882-09 PMC2825926

[69] Sadziene A, Wilske B, Ferdows MS, Barbour AG (1993) The cryptic *ospC* gene of *Borrelia burgdorferi* B31 is located on a circular plasmid. Infect Immun 61: 2192–2195. 8478109 10.1128/iai.61.5.2192-2195.1993 PMC280820

[70] Elias AF, Stewart PE, Grimm D, Caimano MJ, Eggers CH, et al (2002) Clonal polymorphism of *Borrelia burgdorferi* strain B31 MI: implications for mutagenesis in an infectious strain background. Infect Immun 70: 2139–2150. 11895980 10.1128/IAI.70.4.2139-2150.2002 PMC127854

[71] Carroll JA, Garon CF, Schwan TG (1999) Effects of environmental pH on membrane proteins in *Borrelia burgdorferi* . Infect Immun 67: 3181–3187. 10377088 10.1128/iai.67.7.3181-3187.1999 PMC116493

[72] Akins DK, Bourell KW, Caimano MJ, Norgard MV, Radolf JD (1998) A new animal model for studying Lyme disease spirochetes in a mammalian host-adapted state. J Clin Invest 101: 2240–2250. 9593780 10.1172/JCI2325 PMC508812

[73] Ojaimi C, Brooks C, Casjens S, Rosa P, Elias A, et al (2003) Profiling temperature-induced changes in *Borrelia burgdorferi* gene expression using whole genome arrays. Infect Immun 71: 1689–1705. 12654782 10.1128/IAI.71.4.1689-1705.2003 PMC152086

[74] Revel AT, Talaat AM, Norgard MV (2002) DNA microarray analysis of differential gene expression in *Borrelia burgdorferi*, the Lyme disease spirochete. Proc Natl Acad Sci USA 99: 1562–1567. 11830671 10.1073/pnas.032667699 PMC122230

[75] Tokarz R, Anderton JM, Katona LI, Benach JL (2004) Combined effects of blood and temperature shift on *Borrelia burgdorferi* gene expression as determined by whole genome DNA array. Infect Immun 72: 5419–5432. 15322040 10.1128/IAI.72.9.5419-5432.2004 PMC517457

[76] Winter RB, Morrissey L, Gauss P, Gold L, Hsu T, et al (1987) Bacteriophage T4 regA protein binds to mRNAs and prevents translation initiation. Proc Natl Acad Sci U S A 84: 7822–7826. 3120177 10.1073/pnas.84.22.7822 PMC299406

[77] Vytvytska O, Moll I, Kaberdin VR, von Gabain A, Blasi U (2000) Hfq (HF1) stimulates ompA mRNA decay by interfering with ribosome binding. Genes Dev 14: 1109–1118. 10809669 PMC316587

[78] Baker CS, Morozov I, Suzuki K, Romeo T, Babitzke P (2002) CsrA regulates glycogen biosynthesis by preventing translation of *glgC* in *Escherichia coli* . Mol Microbiol 44: 1599–1610. 12067347 10.1046/j.1365-2958.2002.02982.x

[79] Hayes B, Jewett M, Rosa P (2010) A *lacZ* reporter system for use in *Borrelia burgdorferi* . Appl Environ Microbiol 76: 7407–7412. 20851957 10.1128/AEM.01389-10 PMC2976203

[80] Smith AH, Blevins JS, Bachlani GN, Yang XF, Norgard MV (2007) Evidence that RpoS (sigmaS) in *Borrelia burgdorferi* is controlled directly by RpoN (sigma54/sigmaN). J Bacteriol 189: 2139–2144. 17158681 10.1128/JB.01653-06 PMC1855718

[81] Archambault L, Linscott J, Swerdlow N, Boyland K, Riley E, et al (2013) Translational efficiency of *rpoS* mRNA from *Borrelia burgdorferi:* effects of the length and sequence of the mRNA leader region. Biochem Biophys Res Commun 433: 73–78. 23454119 10.1016/j.bbrc.2013.02.063 PMC3616328

[82] Stevenson B, Schwan TG, Rosa PA (1995) Temperature-related differential expression of antigens in the Lyme disease spirochete, *Borrelia burgdorferi* . Infect Immun 63: 4535–4539. 7591099 10.1128/iai.63.11.4535-4539.1995 PMC173648

[83] Sze CW, Li C (2011) Inactivation of *bb0184,* which encodes carbon storage regulator A, represses the infectivity of *Borrelia burgdorferi* . Infect Immun 79: 1270–1279. 21173314 10.1128/IAI.00871-10 PMC3067481

[84] Salman-Dilgimen A, Hardy PO, Dresser AR, Chaconas G (2011) HrpA, a DEAH-box RNA helicase, is involved in global gene regulation in the Lyme disease spirochete. PLoS One 6: e22168. 21814569 10.1371/journal.pone.0022168 PMC3144200

[85] Lease RA, Belfort M (2000) A trans-acting RNA as a control switch in *Escherichia coli*: DsrA modulates function by forming alternative structures. Proc Natl Acad Sci U S A 97: 9919–9924. 10954740 10.1073/pnas.170281497 PMC27626

[86] Majdalani N, Cunning C, Sledjeski D, Elliott T, Gottesman S (1998) DsrA RNA regulates translation of RpoS message by an anti-antisense mechanism, independent of its action as an antisilencer of transcription. Proc Natl Acad Sci U S A 95: 12462–12467. 9770508 10.1073/pnas.95.21.12462 PMC22853

[87] Ades SE (2008) Regulation by destruction: design of the sigmaE envelope stress response. Curr Opin Microbiol 11: 535–540. 18983936 10.1016/j.mib.2008.10.004

[88] Gottesman S (1996) Proteases and their targets in *Escherichia coli* . Annu Rev Genet 30: 465–506. 8982462 10.1146/annurev.genet.30.1.465

[89] Young JC, Hartl FU (2003) A stress sensor for the bacterial periplasm. Cell 113: 1–2. 12679025 10.1016/s0092-8674(03)00192-2

[90] Hengge R (2009) Proteolysis of sigmaS (RpoS) and the general stress response in *Escherichia coli* . Res Microbiol 160: 667–676. 19765651 10.1016/j.resmic.2009.08.014

[91] Studemann A, Noirclerc-Savoye M, Klauck E, Becker G, Schneider D, et al (2003) Sequential recognition of two distinct sites in sigma(S) by the proteolytic targeting factor RssB and ClpX. EMBO J 22: 4111–4120. 12912910 10.1093/emboj/cdg411 PMC175800

[92] Lin T, Gao L, Zhang C, Odeh E, Jacobs MB, et al (2012) Analysis of an ordered, comprehensive STM mutant library in infectious *Borrelia burgdorferi*: insights into the genes required for mouse infectivity. PLoS One 7: e47532. 23133514 10.1371/journal.pone.0047532 PMC3485029

[93] Chen L, Xu Q, Tu J, Ge Y, Liu J, et al (2013) Increasing RpoS Expression Causes Cell Death inBorrelia burgdorferi. PLoS One 8: e83276. 24358270 10.1371/journal.pone.0083276 PMC3865164

[94] Pruteanu M, Hengge-Aronis R (2002) The cellular level of the recognition factor RssB is rate-limiting for sigmaS proteolysis: implications for RssB regulation and signal transduction in sigmaS turnover in *Escherichia coli* . Mol Microbiol 45: 1701–1713. 12354235 10.1046/j.1365-2958.2002.03123.x

[95] Caimano MJ, Kenedy MR, Kairu T, Desrosiers DC, Harman M, et al (2011) The hybrid histidine kinase Hk1 is part of a two-component system that is essential for survival of *Borrelia burgdorferi* in feeding *Ixodes scapularis* ticks. Infect Immun 79: 3117–3130. 21606185 10.1128/IAI.05136-11 PMC3147546

[96] Rogers EA, Terekhova D, Zhang HM, Hovis KM, Schwartz I, et al (2009) Rrp1, a cyclic-di-GMP-producing response regulator, is an important regulator of *Borrelia burgdorferi* core cellular functions. Mol Microbiol 71: 1551–1573. 19210621 10.1111/j.1365-2958.2009.06621.x PMC2843504

[97] Bono JL, Elias AF, Kupko III JJ, Stevenson B, Tilly K, et al (2000) Efficient targeted mutagenesis in *Borrelia burgdorferi* . J Bacteriol 182: 2445–2452. 10762244 10.1128/jb.182.9.2445-2452.2000 PMC111306

[98] Elias AF, Bono JL, Carroll JA, Stewart P, Tilly K, et al (2000) Altered stationary phase response in a *Borrelia burgdorferi rpoS* mutant. J Bacteriol 182: 2909–2918. 10781562 10.1128/jb.182.10.2909-2918.2000 PMC102002

[99] Rego RO, Bestor A, Rosa PA (2011) Defining the plasmid-encoded restriction-modification systems of the Lyme disease spirochete *Borrelia burgdorferi* . J Bacteriol 193: 1161–1171. 21193609 10.1128/JB.01176-10 PMC3067601

[100] Dulebohn DP, Bestor A, Rego RO, Stewart PE, Rosa PA (2011) The *Borrelia burgdorferi* linear plasmid lp38 is dispensable for completion of the mouse-tick infectious cycle. Infect Immun 79: 3510–3517. 21708994 10.1128/IAI.05014-11 PMC3165476

[101] Kawabata H, Norris SJ, Watanabe H (2004) BBE02 disruption mutants of Borrelia burgdorferi B31 have a highly transformable, infectious phenotype. Infect Immun 72: 7147–7154. 15557639 10.1128/IAI.72.12.7147-7154.2004 PMC529111

[102] Barbour AG, Hayes SF, Heiland RA, Schrumpf ME, Tessier SL (1986) A *Borrelia*-specific monoclonal antibody binds to a flagellar epitope. Infect Immun 52: 549–554. 3516878 10.1128/iai.52.2.549-554.1986 PMC261035

[103] Bestor A, Rego RO, Tilly K, Rosa PA (2012) Competitive advantage of *Borrelia burgdorferi* with outer surface protein BBA03 during tick-mediated infection of the mammalian host. Infect Immun 80: 3501–3511. 22851744 10.1128/IAI.00521-12 PMC3457558

[104] Kobryn K, Naigamwalla DZ, Chaconas G (2000) Site-specific DNA binding and bending by the *Borrelia burgdorferi* Hbb protein. Mol Microbiol 37: 145–155. 10931312 10.1046/j.1365-2958.2000.01981.x

