# Supplementary figures and images for: Global Repression of Host-Associated Genes of the Lyme Disease Spirochete through Post-Transcriptional Modulation of the Alternative Sigma Factor RpoS
Source: PLoS One. 2014 Mar 26;9(3):e93141. doi: 10.1371/journal.pone.0093141 (PMC3966842; doi:10.1371/journal.pone.0093141)

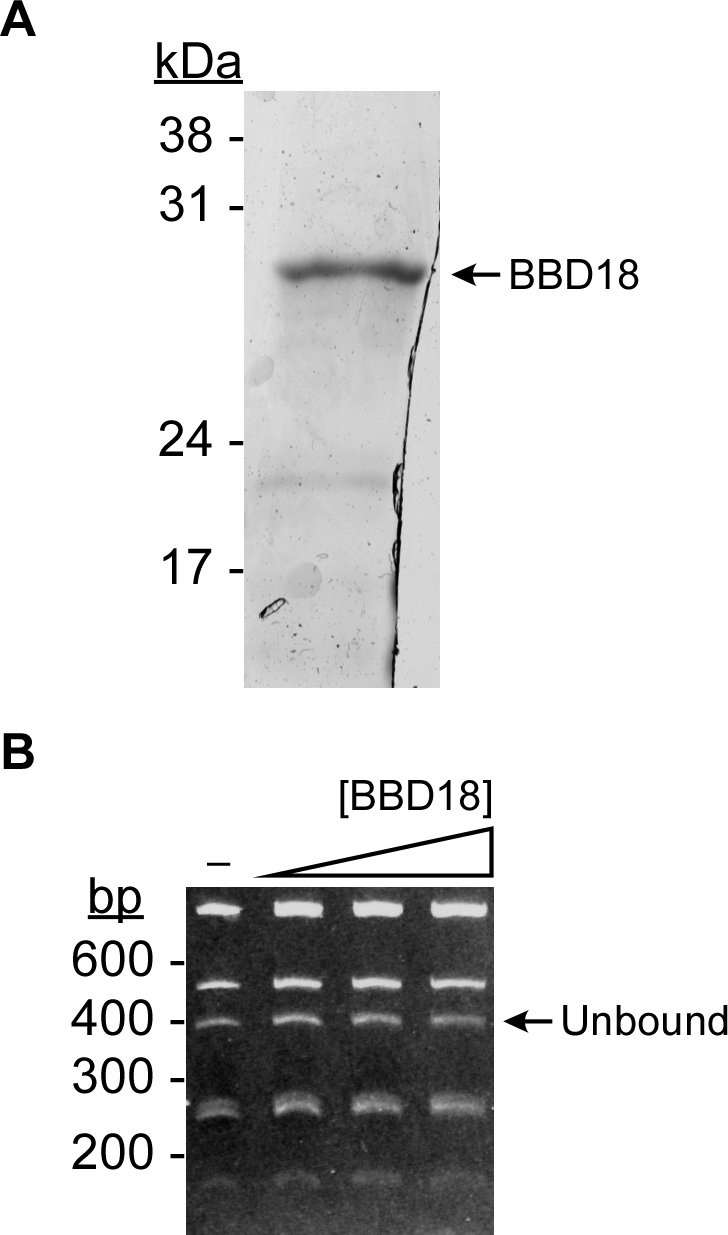

Supplement: Figure S1 — Analysis of a potential BBD18-ospC promoter interaction. Recombinant BBD18 was purified by affinity chromatography and analyzed by SDS-PAGE and Coomassie blue staining (A). Electrophoretic mobility shift assay of restriction digested pPCR8-ospCp-5F, containing the ospC promoter and upstream inverted repeats, was incubated with purified recombinant BBD18 and resolved on a 5% polyacrylamide gel (B). The arrow indicating unbound DNA is directed at the specific restriction fragment containing the ospC promoter and upstream inverted repeats. (TIF) [file pone.0093141.s001.tif]
