# Supplementary material for: Global Repression of Host-Associated Genes of the Lyme Disease Spirochete through Post-Transcriptional Modulation of the Alternative Sigma Factor RpoS
Source: PLoS One. 2014 Mar 26;9(3):e93141. doi: 10.1371/journal.pone.0093141 (PMC3966842; doi:10.1371/journal.pone.0093141)
Supplement: Table S1 — Oligonucleotide primers and probes used in this study. (DOCX) [file pone.0093141.s002.docx]

**Supplementary Table 1**

**Table S1**

|  | Primer Name | Sequence (5'-3')^a^ |  |
| --- | --- | --- | --- |
|  |  |  |  |
| 1 | 5' Gent(*aacc1*) Screen | ATGTTACGCAGCAGCAACGATGTTACGCAGCAGG |  |
| 2 | 3' Gent(*aacc1*) Screen | TTAGGTGGCGGTACTTGGGTCGATATCAAAGTGCATCACTTCTTC |  |
| 3 | 5' *rpoS*-BspHI | ATCATGAACATATTTAGTAATGAGGATTTAAAC |  |
| 4 | 3' *rpo*S-SacI | TTGAGCTCTTAATTTATTTCTTCTTTTAATTTTTTAAG |  |
| 5 | 5'BBD18-Nde1 | AAGATATGCAAAAAGAAATAACAATAAACTATAATGAATATACAATTGGG |  |
| 6 | 3' BBD18 Xho | TTCTCGAGTTAAATTTTGGTTTTTTCCCCATGATTTCCTTTTAATTTTTTAG |  |
| 7 | 5' *rpoS*141p-SalI | AAGTCGACAAAATACTCCCCCTAAACTCAAAATTATATCC |  |
| 8 | 3'*rpoS*141p-BspHI | TTCATGATTATTATATTTTCTCCCCTTTCTTGAATATTG |  |
| 9 | 5' KAN screen | ATATTCAACGGGAAACGTCGAGGCCGCGATTAAATTC |  |
| 10 | 3' KAN screen | TTAGAAAAACTCATCGAGCATCAAATGAAACTGCAATTTATTCATATCAGGATTATC |  |
|  |  |  |  |
|  | |  |  |
| Taqman Primers and probes^b^ | | Sequence (5'-3') |  |
|  |  |  |  |
|  | *flaB* forward | CAGCTAATGTTGCAAATCTTTTCTCT |  |
|  | *flaB* reverse | CCTTCCTGTTGAACACCCTCTT |  |
|  | *flaB* probe | ACTGCTCAGGCTGCACCGGT |  |
|  | *ospC* forward | ACGGATTCTAATGCGGTTTTACTT | [[105](#_ENREF_105)] |
|  | *ospC* reverse | CAATAGCTTTAGCAGCAATTTCATCT | [[105](#_ENREF_105)] |
|  | *ospC* probe | CTGTGAAAGAGGTTGAAGCGTTGCTGTCAT | [[105](#_ENREF_105)] |
|  | *bba66* forward | CTGCTTCTGGTGTGTTAGAGTT |  |
|  | *bba66* reverse | GCTAGATGTTATTTCAAGTGCTAAAG |  |
|  | *bba66* probe | TTATGACAGAAGTTACGCGCCACAGC |  |
|  | *rpoS* forward | AAGGCAACTTGGGATTAATAA |  |
|  | *rpoS* reverse | TACCAATCTGGTTTTAGTGTTTAATG |  |
|  | *rpoS* probe | ATGCATCATTTTGGATTAAGCAATCACT |  |
|  | *bba62* forward | TATTTTTGTCTGGCATTGACT |  |
|  | *bba62* reverse | TTCAGATGAGATGGAAAATACTAG |  |
|  | *bba62* probe | AAGATTCAAAAGTTACAGCTCCAATGACAG |  |
|  | *bba72* forward | TTCTAATTTTGAGACCTTAGTTTCTTCTTG |  |
|  | *bba72* reverse | GGCACTTCATGTGCTGTTAATCC |  |
|  | *bba72* probe | AAAGCCGCACCGATATAAAAGAAAGCAACC |  |
|  | *rpoN* forward | CGTTTGGGTCTTTAAATTCG |  |
|  | *rpoN* reverse | AACACGGCTTTAGAAATTATCA |  |
|  | *rpoN* probe | AAAACTTAACCCCAACCCAACGC |  |
|  | *bbj24* forward | AAGATGATAATGTTCCAGCGGA |  |
|  | *bbj24* reverse | CAATCCTTTCCTATTGTGCGG |  |
|  | *bbj24* probe | AGCAAGCTCTCCCAATGCCCTAAA |  |
|  | *bbg01* forward | GAACTTGAGCAGCTTATAAATATGATTG |  |
|  | *bbg01* reverse | AATAATACTTTCTTTTAACCTTTTCTGAGC |  |
|  | *bbg01* probe | AGATCTGCGGCTTTCTTTTTTGACACC |  |
|  | *bba34* forward | TTGTCTTTGGTGTTGGAATTGG |  |
|  | *bba34* reverse | GCCTGTATCCACCCGTTTTG |  |
|  | *bba34* probe | TGTGGATCAAGCGATGTTGGTTCGT |  |
|  | *bbj41* forward | AACGCAACCATTGACGCTTAC |  |
|  | *bbj41* reverse | GGTTTAGTAGATAAAGAGGATGGGAATATT |  |
|  | *bbj41* probe | ATAATAAAGCGCTAGCAAAACACATAAAGGAT |  |
|  | *bba15* forward | ATGTTAGCAGCCTTGACGAG |  |
|  | *bba15* reverse | CAGAAGTTCCTTTAAGCTCAAGC |  |
|  | *bba15* probe | ACAGCGTTTCAGTAGATTTGCCTGGT |  |
|  |  |  |  |
| a. | Relevant restriction enzyme recognition sites have been underlined | |  |
| b. | Probes are labeled with a 5' flourophore (FAM) and a 3' quencher (TAMRA) | |  |
|  |  |  |  |
|  |  |  |  |
|  |  |  |  |
|  |  |  |  |
|  |  |  |  |
